# *SMAD4 *- Molecular gladiator of the TGF-β signaling is trampled upon by mutational insufficiency in colorectal carcinoma of Kashmiri population: an analysis with relation to *KRAS *proto-oncogene

**DOI:** 10.1186/1471-2407-10-300

**Published:** 2010-06-17

**Authors:** A Syed Sameer, Nissar A Chowdri, Nidda Syeed, Mujeeb Z Banday, Zaffar A Shah, Mushtaq A Siddiqi

**Affiliations:** 1 Department of Immunology and Molecular Medicine, Sher-I-Kashmir Institute of Medical Sciences, Soura, Srinagar, Kashmir, India. 190011; 2 Department of Clinical Biochemistry, Sher-I-Kashmir Institute of Medical Sciences, Soura, Srinagar, Kashmir, India. 190011; 3 Department of General Surgery, Sher-I-Kashmir Institute of Medical Sciences, Soura, Srinagar, Kashmir, India. 190011; 4 Department of Biotechnology, Kashmir University, Hazratbal, Srinagar, Kashmir, India. 190006

## Abstract

**Background:**

The development and progression of colorectal cancer has been extensively studied and the genes responsible have been well characterized. However the correlation between the *SMAD4 *gene mutations with *KRAS *mutant status has not been explored by many studies so far. Here, in this study we aimed to investigate the role of *SMAD4 *gene aberrations in the pathogenesis of CRC in Kashmir valley and to correlate it with various clinicopathological variables and *KRAS *mutant genotype.

**Methods:**

We examined the paired tumor and normal tissue specimens of 86 CRC patients for the occurrence of aberrations in MCR region of *SMAD4 *and exon 1 of *KRAS *by PCR-SSCP and/or PCR-Direct sequencing.

**Results:**

The overall mutation rate of mutation cluster region (MCR) region of *SMAD4 *gene among 86 patients was 18.6% (16 of 86). 68.75% (11/16) of the *SMAD4 *gene mutants were found to have mutations in *KRAS *gene as well. The association between the *KRAS *mutant genotype with *SMAD4 *mutants was found to be significant (P =< 0.05). Further more, we found a significant association of tumor location, tumor grade, node status, occupational exposure to pesticides and bleeding PR/Constipation with the mutation status of the *SMAD4 *gene (P =< 0.05).

**Conclusion:**

Our study suggests that *SMAD4 *gene aberrations are the common event in CRC development but play a differential role in the progression of CRC in higher tumor grade (C+D) and its association with the *KRAS *mutant status suggest that these two molecules together are responsible for the progression of the tumor to higher/advanced stage.

## Background

Colorectal carcinoma (CRC) is a common disease in both men and women worldwide. CRC is the third most common cause of cancer-related death in the western world and so is its incidence in Kashmir [1,2]. The majority of CRCs develop from benign pre-neoplastic lesions: the adenomatous polyps or adenomas. Progression from a benign adenoma to a malignant carcinoma passes through a series of well-defined histological stages, which is referred to as the adenoma-carcinoma sequence [3]. Two major mechanisms of genomic instability have been identified that give rise to colorectal carcinoma development and progression: chromosomal instability (CIN) and microsatellite instability (MIN). CIN is associated with a series of genetic changes that involve the activation of oncogenes as *KRAS *and inactivation of tumor suppressor genes as *TP53*, *SMAD4/DPC4 *and *APC*, while as MIN is associated with mutations in DNA mismatch repair (MMR) genes which affect DNA replication in repetitive sequences (microsatellites), resulting in an accumulation of frameshift mutations in genes that contain microsatellites [4-6].

The discovery of human homologues of the Drosophila *Mad *gene, called *SMAD *genes [7], has been a milestone for understanding the genetics of the CRC whether of familial origin or sporadic, that has opened the Pandora's Box for both developmental and cancer biologists. Mutations in two Smad family member genes - *SMAD4 *(also known as *MADH4*, *DPC4 *&*JIP*) and *SMAD2 *(also known *MADR2*, and *hMAD-2*) have been identified in human cancers and more importantly with high frequency in pancreatic and CRCs [8]. This raises the possibility that one or more of these genes can act as tumor suppressors as well as developmental regulators. Approximately 50% of pancreatic carcinomas, 20% of colon carcinomas, and 10% of lung cancers exhibit mutations in *SMAD4*, and mutations in *SMAD2 *have been found in ~7% of colorectal and lung cancers [7,9].

*SMAD4 *gene is located on the long arm (q) of chromosome 18 at band 21.1. The gene encompasses 49.5 kb of DNA with 13 exons, the first two constituting 5'-UTR as they do not code for any protein. *SMAD4 *mRNA transcript constitutes 3220 nucleotides [10]. The protein of *SMAD4 *gene - Smad4 belongs to the Darfwin family of proteins which harbors two conserved amino- and carboxyl-terminal domains known as MH1 and MH2, respectively. Smad4 in the basal state is found mostly as a homo-oligomer, most likely a trimer. It is ubiquitously expressed within the human body. Smad4 is an intracellular mediator of TGF-β family and activin type 1 receptor. Smad4 mediate TGF-β signaling to regulate cell growth and differentiation. TGF-β stimulation leads to phosphorylation and activation of Smad2 and Smad3, which form complexes with Smad4 that accumulate in the nucleus and regulate transcription of target genes. By interacting with DNA-binding proteins, Smad complexes then positively or negatively regulate the transcription of target genes [11-13].

Considering the important role of *SMAD4 *gene in the colorectal carcinogenesis, we devised our study analyze the role of *SMAD4 *gene aberrations in the pathogenesis of CRC in Kashmir valley and correlate it with various clinicopathological variables and *KRAS *mutant genotype.

## Methods

### Patients and specimens

Out of 124 patients who were diagnosed with colorectal carcinoma (CRC) by clinicians using either sigmoidscopy or colonoscopy and confirmed by MRI, a total of 86 colorectal cancer tissue specimens comprising tumor tissues and the corresponding adjacent normal tissues as controls were collected for analysis in this study. All samples were surgically resected and were collected fresh at the Department of Surgery of Sher-I-Kashmir Institute of Medical Sciences, Srinagar, Kashmir. Tissue samples were divided into two parts; one part was sent to histopathological diagnosis and other half was stored at -70°C for molecular investigations. Only histopathologically confirmed cases were included for molecular analysis. The specimens (both tumor and adjacent normal) were snap-frozen at -70°C immediately until further analysis. No follow up of the CRC patients was carried out after the curative surgery. Written informed consent was obtained from all the subjects (and or guardian) included in the study was taken on pre-designed questionnaire (Available on request) and was carried out in accordance with the principles of the Helsinki Declaration. The study protocol was approved by the Research Ethics Committee of Sher-I-Kashmir Institute of Medical Sciences, Kashmir.

### DNA isolation

Genomic DNA was extracted from blood and tissue samples (previously stored in -70°C) of colorectal cancer patients using DNA Extraction Kit II (Zymo Research, USA) for examining mutations in MCR region (exon 2, 8-11) of *SMAD4 *gene and exon 1 of *KRAS *gene. The tissue for DNA extraction from tumor sample was chosen by experienced pathologist and was ascertained to contain more than 90% of the tumor cells.

### PCR-SSCP analysis

The *SMAD4 *and *KRAS *gene analysis was done on all the extracted DNA samples. The MCR region of *SMAD4 *gene comprising of exons 2, 8, 9, 10 and 11 and exon 1 of *KRAS *were amplified using specific oligonucleotide primers [Table 1] [Additional file 1]. PCR was performed in a 25 μl total volume reaction mixture containing 50 ng of genomic DNA, 100 ng of each primer, 100 μM of each dNTP, 1.5 mM MgCl_2_, 10× of Taq buffer and 1 U of Taq DNA polymerase (Genie, India). The conditions of PCR were as: initial denaturation at 95°C for 5 min, 35 cycles of denaturation at 95°C, annealing at X°C [Table 1] and extension at 72°C, for 30 seconds each and final extension at 72°C for 7 min in Biorad *i*cycler. The PCR products were run on 2% agarose gel and analyzed under UV illuminator.

**Table 1:** Specific primers for *SMAD4 *mutational analysis

*Gene/Exon*	*Sequence*	*Amplicon Size (bp)*	***T***_***m ***_***(***^***o***^***C)***
***SMAD4/Ex 2***	F 5'-TTC TAGGTG GCT GGT CGG AA-3' R 5'-CAG GTG ATA CAA CTC GTT CG-3'	175	56

***SMAD4/Ex 8***	F 5'-TTT CTC ATG GGA GGA TGT TC-3' R 5'-CAA TTT TTT AAA GTA ACT ATC TGA C-3'	264	57

***SMAD4/Ex 9***	F 5'- TAT TAA GCA TGC TAT ACA ATC TG -3' R 5'- CTT CCA CCC AGA TTT CAA TTC -3'	332	58

***SMAD4/Ex 10***	F 5'-GAA TTT TCT TTA TGA ACT CAT AG-3' R 5'-TTT AAA AAA GAA TGA AAA GCA TAC-3'	213	57

***SMAD4/Ex 11***	F 5'-CTG ATG TCT TCC AAA CTC TTT TCT G-3' R 5'-TGT ATT TTG TAG TCC ACC ATC-3'	299	57

***KRAS/Ex1***	F: 5'-CTG CTG AAA ATG ACT GAA TA-3' R: 5'-ATG GTC CTG CAC CAG TAA TA-3'	162	48

Yakicier MC et al; Oncogene. 1999: 18: 4879-83.

Hadžija MP et al; Mutation Research. 2004; 548: 61-73.

Sameer AS et al; Saudi J Gastroenterol. 2009; 15: 244-252.

SSCP analysis of PCR product was carried out on 6% non-denaturing Polyacrylamide gel (PAG) utilizing either non-radioactive silver staining or radioactive procedures [14-16] [Additional file 1]. In non-radioactive SSCP analysis, PCR products mixed in denaturing buffer (95% formamide, 10 mM NaOH, 0.05% xylene-cyanol FF and 0.05% bromophenol blue) in 1:1 ratio were heat denatured at 95°C for 5 min, immediately cooled on ice for 20 min, 6 μl of which were loaded on 6% PAG and eletrophoresed in 0.5× Tris-borate EDTA buffer at ± 17°C at 4 W constant power for 18-22 h. Gels were then silver stained. In radioactive SSCP analysis, radio labeled PCR products (using α32-pCTP) mixed in denaturing loading buffer (95% formamide, 20 mM EDTA, 0.05% xylene-cyanol FF and 0.05% (bromophenol blue) in 1:10 ratio were heat denatured at 95°C for 5 min, 3 μl of which were loaded on 6% PAG and electrophoresed at 4 W in 0.5× Tris-borate EDTA buffer at ± 17°C for 18-22 h. Gel was then transferred onto 3 mm Whatmann paper, covered with saran wrap and dried in vacuum drier at 90°C for 1 h. The saran wrap was then replaced by X-ray film and kept at -70°C for 48 h.

The mobility shift in DNA bands were visualized by developing the X-ray film in a developer. Purified PCR products of the samples showing mobility shift on SSCP analysis and randomly chosen samples were used for direct DNA sequencing.

### Direct Sequencing

PCR amplicons of the tumor samples and from randomly chosen normal samples were first purified by DNA recovery kit (Zymo Research, USA) and then used for direct DNA sequencing. DNA sequencing was carried out at *MACROGEN INC, Korea*. To minimize the sequencing artifacts by PCR, amplicons from at least two different PCRs were sequenced using forward and reverse primers.

### Statistical Analysis

All statistical analysis was performed using PASW, Version 18 (IBM, USA). Pearson's chi-square two-proportion test was used to evaluate the hypothesis of equal distribution of molecular alterations in different clinic-pathological variables. A chi-square probability (P values) of 0.05 or less was considered to be statistically significant. No adjustment was made for performing multiple tests.

## Results

Out of 86 confirmed cases of CRC, 38 were of Duke's A+B Stage and 48 were of Stage C+D. All of them presented constipation and bleeding per rectum as their chief complaint. Further more, out of 86 confirmed cases of CRC, 81 cases were sporadic, 4 were FAP and one case was Lynch Syndrome. All but one case were adenocarcinoma and only one was squamous cell carcinoma (SCC) of basal cell type. 37 cases were females and 49 males, 59 rural and 27 urban, 36 cases had carcinoma in colon and 50 in rectum and 55 were smokers and 31 non smokers [Table 2].

**Table 2:** Nature of *SMAD4 *MCR region mutations in 86 colorectal carcinoma patients from Kashmir valley

*Patient ID*	***Age/Sex***^***a***^	***Dwelling***^***b***^	***Duke's Stage***^***c***^	***Smoking Status***^***d***^	***Node Status***^***e***^	***Site***^***f***^	***Nature***^***g***^	*Pesticide Exposure*	*SMAD4 Exon*	***Mutation***^***h***^	*Amino Acid Change*	*Affected Codon*	***Effect***^***i***^	*KRAS Status/Affected Codon*
X01	55/M	R	A	NSk	N	C	LS	Y	2	TGT > *C*GT	Cys > Arg	115	MS	M; 12 Gly > Asp

X06	28/M	R	C	Sk	Y	R	S	Y	8	CGC > *A*GC	Arg > Ser	361	MS	W

X12	65/F	R	C	Sk	Y	C	S	Y	10	CAG > *T*AG	Gln > Stop	442	NS	W

X16	62/F	U	C	Sk	Y	C	S	N	8	CGC > C*A*C	Arg > His	361	MS	W

X17	67/F	R	D	Sk	Y	R	S	Y	8	TTT>TT*G*	Phe > Leu	362	MS	M; 12 Gly > Asp

X30	44/F	R	D	Sk	Y	C	S	Y	8	TGT > *A*GT	Cys > Ser	363	MS	M; 12 Gly > Asp

X31	64/M	R	C	Sk	Y	C	FAP	Y	8	GGT > G*A*T	Gly > Asp	341	MS	W

X43	80/F	U	D	Sk	Y	R	S	Y	10	GCT>GC*C*	Ala >Ala	456	S	W

X47	60/F	R	B	Sk	N	C	S	Y	11	AAAGGC > AA*TT*GC	Lys > Asn; Gly > Cys	507/8	MS	M; 13 Gly > Cys

X51	58/M	R	C	Sk	Y	C	S	Y	11	GGC > *A*GC	Gly > Ser	508	MS	M; 12 Gly > Ser

X56	45/M	U	D	Sk	Y	R	S	Y	9	TGG > *G*GG	Trp > Gly	419	MS	M; 19 Leu > Phe

X57	40/M	R	D	Sk	Y	C	FAP	N	9	*AGACA*GAG > AGAG	Deletion	415/16 <<	FS	M; 16 Lys > Stop

X65	43/M	R	C	NSk	Y	C	S	Y	8	CGC > C*A*C	Arg > His	361	MS	M; 19 Leu > Phe

X75	55/F	R	C	NSk	Y	C	S	Y	11	AAA > *C*AA	Lys > Gln	507	MS	M; 13 Gly > Arg

X85	38/M	U	C	Sk	Y	C	S	N	9	AGA > A*A*A	Arg > Lys	415	MS	M; 16 Lys > Stop

X86	60/M	R	D	Sk	Y	C	S	Y	10	CGA > *T*GA	Arg > Stop	445	NS	M; 12 Gly > Ala

^**a**^**Age/Sex**: M = Male, F = Female; ^**b**^**Dwelling**: R = Rural, U = Urban; ^**c**^**Duke's stage**: A - Tumor confined to the intestinal wall; B - Tumor invading through the intestinal wall; C - With lymph node(s) involvement; D - With distant metastasis; ^**d**^**Smoking Status**: Sk: Smokers; NSk: Non Smokers; ^**e**^**Node Status**: N = Negative, P = Positive; ^**f**^**Site of tumor**: C = Colon, R = Rectum; ^**g**^**Nature of tumor**: S: Sporadic; LS: Lynch Syndrome; FAP: Familial Adenomatous Polyposis; ^**h**^**Mutation**: Mutated or inserted nucleotide underlined; ^**h**^**Mutation Effect**: MS: Missense mutation; NS: Nonsense mutation; S: Silent mutation; FS: Frameshift mutation.

**Figure 1:**
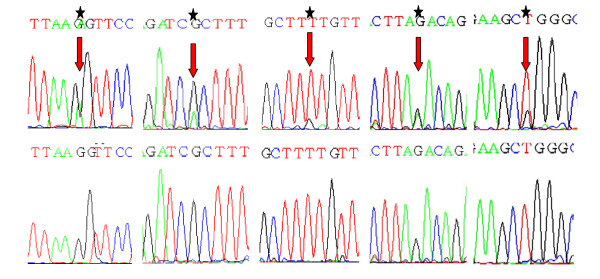
**Partial nucleotide sequence representing the mutant (Above) and normal (Below) {Shown by asterik and arrows}, of MCR region of *SMAD4 *gene**. 1A: Transition of G > A at codon 341; GGT > G*A*T; Gly > Asp in Exon8 of *SMAD4 *gene. 1B: Transition of G > A at codon 361; CGC > C*A*C; Arg > His in Exon8 of *SMAD4 *gene. 1C: Transversion of T > G at codon 362; TTT > TT*G*; Phe > Leu in Exon8 of *SMAD4 *gene. 1D: Transition of G > A at Codon 415; AGA > A*A*A; Arg > Lys in Exon9 of *SMAD4 *gene	1E: Transversion of T > G at codon 419; TGG > *G*GG; Trp > Gly in Exon9 of *SMAD4 *gene

**Figure 2:**
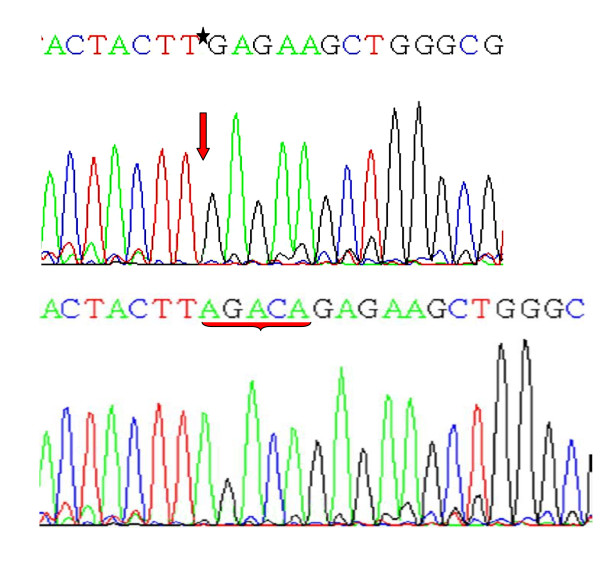
Partial nucleotide sequence representing the mutant (Above) and normal (Below) {Shown by asterik and arrows}, of Deletion of *AGACA *pentamer at codons 415/416 in Exon9 (MCR) of *SMAD4 *gene

**Figure 3:**
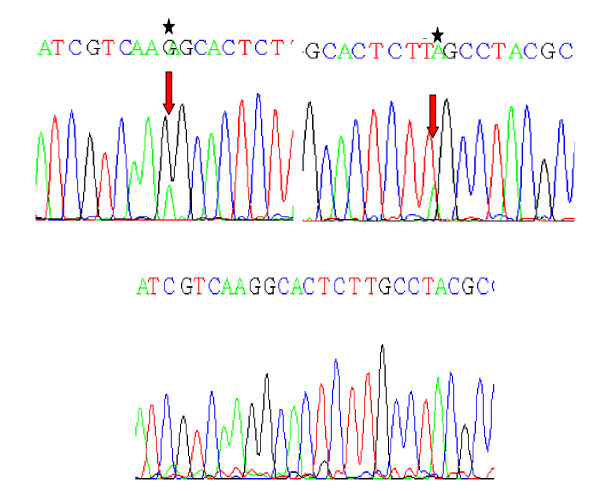
**Partial nucleotide sequence (reverse) representing the mutant (A) and normal (B) {Shown by asterik and arrows}, of *KRAS *exon 1 gene**. 3A: Transition of C > T at codon 19; CTT > *T*TT; Leu > Phe in Exon 1 of *KRAS *oncogene. 3B: Transversion of A > T at codon 16; AAG > TAG; Lys > Stop in Exon 1 of *KRAS *oncogene

**Figure 4:**
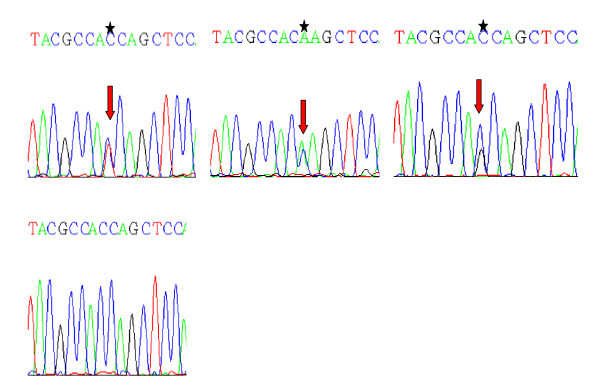
**Partial nucleotide sequence (reverse) representing the mutant (A) and normal (B) {Shown by asterik and arrows}, of *KRAS *exon 1 gene**. 4A: Transition of G > A at codon 12; to GGT > G*A*T; Gly > Asp in Exon 1 of *KRAS *oncogene. 4B: Transversion of G > T at codon 12; GGT > *T*GT; Gly > Cys in Exon 1 of *KRAS *oncogene. 4C: Transversion of G > C at codon 12; GGT > G*C*T; Gly > Ala in Exon 1 of *KRAS *oncogene

The overall mutation rate of mutation cluster region (MCR) of *SMAD4 *gene among 86 patients was 18.6% (16 of 86) [Table 2, Figure 1 &2]. 68.75% (11/16) of the *SMAD4 *gene mutants were found to have mutations in *KRAS *gene as well, the data (of *KRAS *mutations only) has been previously declared by our lab in two studies [14,15] by *Sameer et al*, 2009. Out of 11 *KRAS *mutants seven had mutations that has been reported already by our lab. In addition to those we also found two new novel mutations in *KRAS *oncogene in new procured tumor tissue samples. One was the A:T > T:A transversion of in codon 16 resulting in the truncation (AAG > TAG; Lys > Stop) of Kras protein in two tumor tissues and second was the C:T > T:C transition at codon 19 leading to change of leucine to phenaylalanine (CTT > TTT) in two tumor tissues [Table 2, Figures 3 &4]. In Total 21 of 86 (24.4%) tumor tissues had mutant *KRAS *gene out of these 21, 11 (52.4%) were *SMAD4 *gene mutants [Table 3 &4]. There were 9 (56.25%) tumors which had both *KRAS *activating mutations as well as *SMAD4 *single point mutations. Furthermore, in case of advanced/higher grade tumors (C+D = 48), *KRAS *gene mutations was found 15 (31.25%) and SMAD4 gene was found to be mutated in 14 (29.2%) tumors. Also, 9 (18.75%) of C + D grade tumors had mutations in both *KRAS *as well as *SMAD4 *gene as compared to only 2 (5.2%) of A+B grade tumors [Table 4 &5]. The mutant status of *KRAS *and *SMAD4 *gene was found to be significantly associated with the higher tumor grade (C+D) (P value = 0.03).

**Table 3:** Clinico-epidemiological variables of the 86 colorectal carcinoma patients versus 16 mutant phenotypes of *SMAD4 *gene

Variable	Total N = 86	Mutants M = 16 (18.6%)	P value*
**Age group**			
≤60	52(60.5%)	11(21.2%)	NS
>60	34(39.5%)	5(14.7%)	

**Gender**			
Female	37 (43.0%)	7 (18.9%)	NS
Male	49 (67.0%)	9 (18.4%)	

**Dwelling**			
Rural	59 (68.6%)	12 (20.3%)	NS
Urban	27 (31.4%)	4 (14.8%)	

**Tumor location**			
Colon	36 (41.9%)	12 (33.3%)	***<0.01***
Rectum	50 (58.1%)	4 (8.0%	

**Nodal status**			
Involved	48 (55.8%)	14 (29.2%)	***<0.01***
Not Involved	38 (44.2%)	2 (5.3%)	

**Tumor grade**			
A + B	38 (44.2%)	2 (5.3%)	***<0.01***
C + D	48 (55.8%)	14 (29.2%)	

**Smoking status**			
Ever	55 (64.0%)	13 (23.6%)	NS
Never	31 (36.0%)	3 (9.6%)	

***Bleeding PR/Constipation***			
Yes	60 (69.8%)	15 (25.0%)	***<0.01***
No	26 (30.2%)	1 (3.8%)	

**Pesticide Exposure**			
Ever	53 (61.6%)	13 (24.5%)	***<0.05***
Never	33 (38.4%)	3 (9.1%)	

***KRAS *Status**			
Wild-type	65 (75.6%)	5 (7.7%)	***< 0.01***
Mutated	21 (24.4%)	11 (52.4%)	

*Pearson's two proportion test

**Table 4:** Correlation of *SMAD4 *gene status versus *KRAS *gene status

	*SMAD4 *Status		
	Wild N = 70	Mutant M = 16	*OR; P Value; CI (95%)*
***KRAS *Status**			
Wild; n = 65	60 (92.3%)	5 (7.7%)	0.07; ***0.00003***; 0.021- 0.26
Mutant; n = 21	10 (47.6%)	11(52.4%)	

Analysis of the mutation spectrum of MCR region of *SMAD4 *gene revealed 16 mutations in total. There were twelve missense mutations, two nonsense mutations, one silent mutation and one frameshift mutation - including nine transitions and four transversions. The frame shift mutation was observed in codons 415/416 (exon 9) due to deletion of AGACA pentamer [Table 2, Figure 2]. Among the transistions, G:A > A:G substitutions were most prevalent followed by C:T > T:C. The two nonsense mutations included, CAG > TAG transition leading to Gln > Stop at codon 442 and other CGA > TGA transition leading to Arg > Stop at codon 445, both occurred in exon 10 of *SMAD4 *gene [Table 2, Figure 1].

A number of salient and interesting features were found to be associated with the *SMAD4 *gene mutants, Among the 16 mutations seen, there were one (6.25%) in exon 2, six (37.5%) in exon 8, three (18.75%) each in exon 9, exon 10, and exon 11 of MCR region of *SMAD4 *gene [Table 2]. Clearly the hot spot exon within the MCR region of the *SMAD4 *gene was exon 8 and within the exon 8 it was the codon 361 which was frequently aberrated due to mutations. Out of 16 *SMAD4 *mutants two (X31 & X57) were of FAP nature, one was LS (X01) and 13 remaining were of sporadic nature [Table 2].

Further more, we found a significant association of Tumor location, Nodal status and Bleeding PR/Constipation with the mutation status of the *SMAD4 *gene (P =< 0.05). We also found association between the occupational exposure to pesticides with that of the mutant status of *SMAD4 *gene (P =< 0.05) identifying it as a potential risk factor. There was also a significant association of the *SMAD4 *mutants with *KRAS *mutants [Table 3].

## Discussion

Kashmir valley located in the northern division of India, surrounded by Himalayas has an unique ethnic population living in a temperate environmental conditions having distinctive food habits which play an overwhelming role in the development of GIT cancers over the genetic factors [17-19]. The food habits include consumption of sun-dried and smoked fish and meat, dried and pickled vegetables, red chilly, *Hakh *(a leafy vegetable of *Brassica *family), hot *noon chai *(salted tea) and *Hukka *(water pipe) smoke [20]. As previously reported [21], the etiology and incidence of various GIT cancers in this population has been attributed to a probable exposure to nitroso compounds, amines and nitrates reported to be present in these local food stuffs.

Colorectal cancer being the commonest cancer, is the major cause of mortality and morbidity worldwide, there are nearly one million new cases of colorectal cancer diagnosed world-wide each year and half a million deaths [22]. The incidence of this malignancy shows considerable variation among racially or ethnically defined populations in multiracial/ethnic countries. Kashmir has been reported by now as a high-incidence area of GIT cancers [19,20]. Colorectal Cancer in Kashmir valley is the third most common GIT cancer after esophageal and gastric as per SKIMS Medical record registry, with almost over 124 cases reported by the end of year 2009.

Among the most common genetic abnormalities in colorectal carcinomas, deletions in the long arm of chromosome 18 are the foremost one [3]. A number of different genes have been shown to be the target of these deletions, which include *APC *and *SMAD2 *and *SMAD4 *- the two genes of the TGF-β signal transduction cascade [23]. TGF-β signaling is an important regulator of proliferation, differentiation, and apoptosis with a key role in non-transformed human colon epithelium homeostasis [24,25]. The *SMAD4 *gene - already identified as a candidate tumor suppressor gene for pancreatic cancers [7] located at 18q21.1 in close proximity to *APC*, has recently attracted considerable interest as a prime candidate target gene for the 18q deletions because of recent data linking mutations in this gene to sporadic and familial colorectal cancer [26,27].

TGF-β signaling regulates tumorigenesis and in human cancer its signaling pathways are often modified during tumor progression [28]. The growth inhibitory effect of TGF-β signaling in epithelial cells explains its role as a tumor suppressor in carcinomas, although TGF-β expression by tumor cells contributes to cancer progression as well [29,30] thus unlike other signaling pathways it plays a dual role [31]. Smads are the key intracellular mediators of transcriptional responses to TGF-β signaling, and among these Smad4 is the pivotal factor of the TGF-β pathway as it functions as a key tumor suppressor [24-26,29-31]. The role of Smad4 gene as an important tumor suppressor gene came out by the novel study of the allelotype loss in pancreatic adenocarcinoma [32]. This study showed that about 90% of these tumors show allelic loss of chromosome 18q. In the same year another study identified the genetic target of these allelic losses as the *SMAD4/DPC4 *gene (DPC-Deleted in Pancreatic Carcinoma, locus 4). The study analyzed 338 tumors, originating from 12 distinct anatomic sites, for alterations in the *SMAD4/DPC4 *gene. An alteration of the *SMAD4/DPC4 *gene sequence was identified in one of eight breast carcinomas and one of eight ovarian carcinomas. *SMAD4/DPC4 *was found to be homozygously deleted in about 30% of pancreatic carcinomas and inactivated by intragenic mutation in another 20% of the tumors. The tissue restriction of alterations in *SMAD4/DPC4*, as in many other tumor-suppressor genes, emphasizes the complexity of rate-limiting checkpoints in human tumorigenesis [33]. Most of Smad4 gene mutations in human cancer are missense, nonsense, and frameshift mutations at the mad homology 2 region (MH2) which interfere with the homo-oligomer formation of Smad4 protein and hetero-oligomer formation between Smad4 and Smad2 proteins, resulting in disruption of TGF-β signaling [12,26,33-35].

Our study was carried out to clarify the involvement of *SMAD4 *in the development and progression of colorectal carcinoma where it is reported to be mutated or deleted in about 20% of invasive cases [26] and the relationship between the mutant status of *SMAD4 *and *KRAS *genes. We found that 15 out of 16 mutations of *SMAD4 *gene occurred in C-terminal region of *SMAD4 *gene which codes for MH2 domain of Smad protein. These results are in conformation with the previous studies [26,27,36] carried out on this gene. The MH2 domain of Smad4 protein is a multifunctional region that mediates differential association with a wide variety of proteins. Many of these interactions serve to provide specificity and selectivity to Smad function. The Smads exist as monomers and trimers, and through various studies it is clear that the MH2 domain is critical for mediating interactions in the oligomers [12]. The mutations in this region henceforth are deleterious to the overall interaction of Smad protein with itself and with other downstream proteins, thereby affecting the signal transduction of TGF-β pathway and hence hampering normal colonic mucosal cell development and differentiation. Furthermore, in a recent study by Zhang *et al*, 2009 on transgenic mice [37] it was found beyond doubt that wild smad4 protein played an important anti-metastatic role in the development and progression of CRC. It was also found in this seminal study that loss of Smad4 in MC38 cells plays an important role in increasing the tumorigenic and metastatic potential of these cells as the loss of smad4 protein appears to switch TGF-β from a tumor suppressor to a tumor promoter pathway.

Another salient feature of the mutations found in this study was the deletion of AGACA pentamer in exon 9 of the *SMAD4 *gene in one tumor tissue from a Familial Adenomatous Polyposis case [Figure 2]. This affected codon is 415/16 of the *SMAD4 *gene reflecting its effect on MH2 domain of Smad4 protein. Similar type of deletion has also been reported previously by Miyaki *et al*, 1999 in Familial Adenomatous Polyposis (FAP) tumors within the same region of the *SMAD4 *gene [26]. Also, this tumor tissue was found to be *KRAS *mutant. The two nonsense mutations found in this study - one affecting codon 442 (Gln > Stop) and other codon 445 (Arg > Stop) occurred in exon 10 of *SMAD4 *gene. Both of these mutations lead to the synthesis of truncated Smad4 protein devoid of oligomerisation properties. One of these mutant tumors was found to be *KRAS *mutant at codon 12 (Gly > Ala) as well [Figure 3 &4].

We also assessed the relationship between the TGF-β pathway and MAP Kinase pathway via *SMAD4 *and *KRAS *proteins. We found that 21 out of 86 (24.4%) tumors were *KRAS *mutants, and out of 16 *SMAD4 *mutants 11 (52.4%) were *KRAS *mutants also. The cross talk mechanism between these two pathways has been experimented upon and conceptualized previously [38]. Cross-talk between *RAS *and TGF-β signaling has been reported to play important roles in various physiological and pathological processes, and *RAS *signal has been reported to regulate TGF-β signaling both positively and negatively [39]. *RAS *transformation in lung, intestinal, liver, pancreas, and mammary epithelial cells has been reported to confer resistance to growth inhibition by TGF-β [40-42].

We found a significant association between the mutant statuses of the *SMAD4 *gene with that of the *KRAS *gene, thereby suggesting that similar mechanisms in our population where a cross talk between the MAP Kinase and TGF-β pathways might play a role in the development of CRC to advanced stage cumulatively [38,39,43] as we found 29 (60.4%) of higher grade tumors (C+D) were mutants for either of the two genes (*KRAS *and *SMAD4*) [Table 5]. Also, we found that 9/11 (81.8%) of the both gene mutants belonged to C+D tumor grade category. This observation clearly suggested a link between these two important cell development controlling genes. In other words we can say that the tumor which has *SMAD4 *mutant status is also likely to have *KRAS *gene mutant status. Janda *et al*, 2002 [44] reported that hyperactivation of the Raf-MAP kinase pathway synergizes with TGF-β signaling and induces epithelial-mesenchymal transition (EMT) and accelerates the tumorigenesis and metastasis of the otherwise normal cells. EMT has been found to be an important tread in the invasion and metastasis of cancer and TGF-β prominently induces progression of cancer through EMT [45].

**Table 5:** Correlation of tumor grade versus *SMAD4 *gene status versus *KRAS *gene status

	*Mutants*			
	*KRAS N = 21*	*SMAD4 N = 16*	χ^2^, P value	Both *N = 11*
***Tumor Grade***				
A + B = 38	6 (28.6%)	2 (12.5%)		2 (18.2%)
C + D = 48	15 (71.4%)	14 (87.5%)	6.63, ***0.03***	9 (81.8%)
				OR = 0.28; 95% CI = 0.05-1.37

In a seminal study by Bardeesy et al, 2006 [46] on genetically engineered mice the impact of *SMAD4 *deficiency on the initiation, development and/or progression of pancreatic ductal adenocarcinoma (PDAC) was ascertained. It was observed that selective *SMAD4 *deletion in the pancreatic epithelium had no discernable impact on pancreatic development or physiology, but when combined with the activated *KRAS*^G12D ^allele, *SMAD4 *deficiency enabled rapid progression of *KRAS*^G12D^-initiated neoplasms. The study concluded that *SMAD4 *is a PDAC tumor suppressor, functioning to block the progression of *KRAS*^G12D^-initiated neoplasm's. In another similar study by Izeradjene *et al*, 2007 [47] on various molecular aspects on invasive adenocarcinoma of pancreas, it was revealed that *KRAS*^G12D ^tumors had higher chances of developing the *SMAD4 *haplo-insufficiency, which in turn leads to invasiveness of the tumor.

As reported by many authors, the substitutions of codon 12 and 13 are predominant mutations in *KRAS *gene [48,49]. These amino-acid substitutions in *KRAS *alters its GTPase activity to a different extent and/or its ability to interact with its regulators, depending upon the substituted amino-acid residue [14]. *KRAS*^G12V ^and *KRAS*^G12R ^mutants are aggressive transforming phenotypes, while *KRAS*^G12S ^and *KRAS*^G12D ^have less striking morphological effects [49]. In addition to the commonly reported activating mutation our analysis of *KRAS *gene also revealed a novel transversion of A > T in codon 16 (Lys > Stop) and an activating mutation in codon 19 (Leu to Phe). This study is the first to report these two novel mutations. Similar kind of mutations have been previously reported from this part of globe [50], where codons 15, 18, 20 and 30 of *KRAS *have been implicated in the tumorigenesis of CRC. Wang et al in their study showed that codon 15 *Kras *mutant protein has less GTPase activity than that of the wild-type *Kras *protein due to a lack of response to GAP induction owing to its less sensitivity to GAP.

Further more, we also found the *SMAD4 *mutant status associated with occupational exposure to pesticides, signifying that pesticides serves as the important risk factor in the development of CRC like other cancers [Table 3], as has been previously reported in various studies [51-53]. This finding was also in tune with the study of Lee *et al*, 2007 [54] in which various chemicals present in pesticides were found to be associated with the significant increased risk of colorectal cancer. In a seminal study by Blair *et al*, carried out on pesticide exposure to farmers indicated that farmers were more favorable to die of diseases including all causes combined, heart disease, as well as cancers of the lung, bladder, liver, colon, esophagus, rectum, and kidney. Following the criteria of Teitelbaum SL, 2002 [55], we also identified pesticide exposure as a potential risk factor for the development of CRC in Kashmir valley, since most of the cases reported in this part of globe are from rural areas and more importantly people who are directly or indirectly associated with agriculture as their main occupation and source of lively hood.

## Conclusion

In this novel study, we found that *SMAD4 *gene aberrations are the common event in CRC development but play a differential role in the progression of CRC in higher tumor grade (C + D). We have also observed a statistically significant association of *SMAD4 *gene aberrations with *KRAS *mutant status suggesting the involvement of at least two molecules in the advanced tumor grade in colorectal cancers; in other words, in case of high grade advanced tumor, aberration in more than one molecular gate keeper of several signal transduction pathways may be involved.

## Competing interests

The authors declare that they have no competing interests.

## Authors' contributions

ASS conceived, designed and performed the lab work of the study. NAC procured and provided the tumor samples for the study. MZB and NSS assisted in the lab work of the study. ZAS, SA and MAS coordinated the study and revised the manuscript. All authors read and approved the final manuscript.

## Pre-publication history

The pre-publication history for this paper can be accessed here:

http://www.biomedcentral.com/1471-2407/10/300/prepub

## Supplementary Material

Additional file 1 **Representative gel pictures of all the amplicons of the *SMAD4 *MCR (exon 2, 8-11) and *KRAS *exon 1 and also SSCP gel picture of *KRAS *exon 1 amplicons**. Click here for file
