# Supplementary material for: SMAD4 - Molecular gladiator of the TGF-β signaling is trampled upon by mutational insufficiency in colorectal carcinoma of Kashmiri population: an analysis with relation to KRAS proto-oncogene
Source: BMC Cancer. 2010 Jun 17;10:300. doi: 10.1186/1471-2407-10-300 (PMC2927996; doi:10.1186/1471-2407-10-300)

**Supplement Figure 1A:** Amplified DNA fragments of exon 2 of *SMAD4* (175bp amplicon) gene

Lane M: 100 bp molecular ladder

Lanes 1-6: Amplicons from different tumor tissues

M 1 2 3 4 5 6


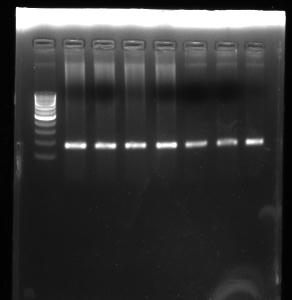


**Supplement Figure 1B:** Amplified DNA fragments of exon 8 of *SMAD4* (264bp amplicon) gene

Lane M: 100 bp molecular ladder

Lanes 1-6: Amplicons from different tumor tissues

M 1 2 3 4 5 6


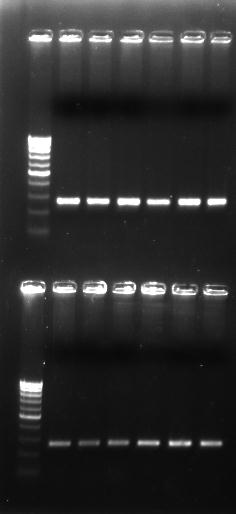


**Supplement Figure 1C:** Amplified DNA fragments of exon 9 of *SMAD4* (264bp amplicon) gene

Lane M: 100 bp molecular ladder

Lanes 1-6: Amplicons from different tumor tissues

M 1 2 3 4 5 6


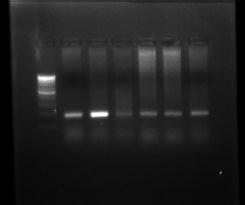


**Supplement Figure 1D:** Amplified DNA fragments of exon 10 of *SMAD4* (213bp amplicon) gene

Lane M: 100 bp molecular ladder

Lanes 1-6: Amplicons from different tumor tissues

M 1 2 3 4 5 6


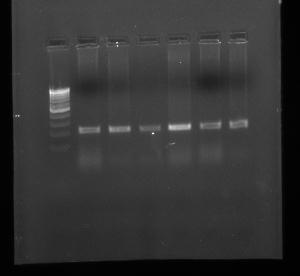


**Supplement Figure 1E:** Amplified DNA fragments of exon 11 of *SMAD4* (299bp amplicon) gene

Lane M: 100 bp molecular ladder

Lanes 1-6: Amplicons from different tumor tissues

M 1 2 3 4 5 6


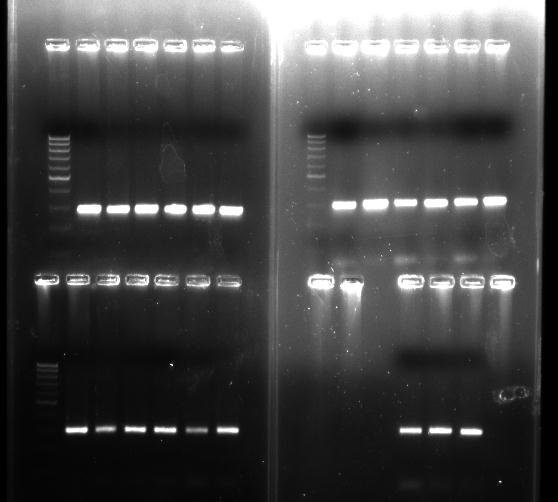


**Supplement Figure 1F:** Amplified DNA fragments of exon 1 of *KRAS* (162bp amplicon) gene

Lane M: 100 bp molecular ladder

Lanes 1-6: Amplicons from different tumor tissues

M 1 2 3 4 5 6


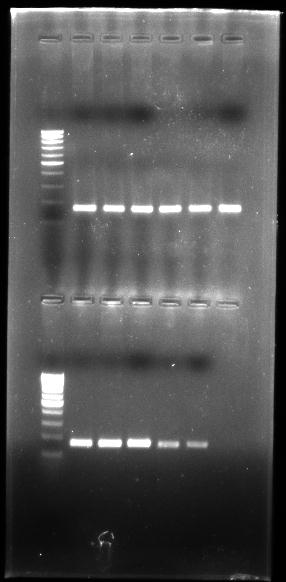


**Supplement Figure 2:** A radioactive SSCP analysis of *KRAS* exon 1 showing mobility shifts in tumor sample T4 (Lane 8)


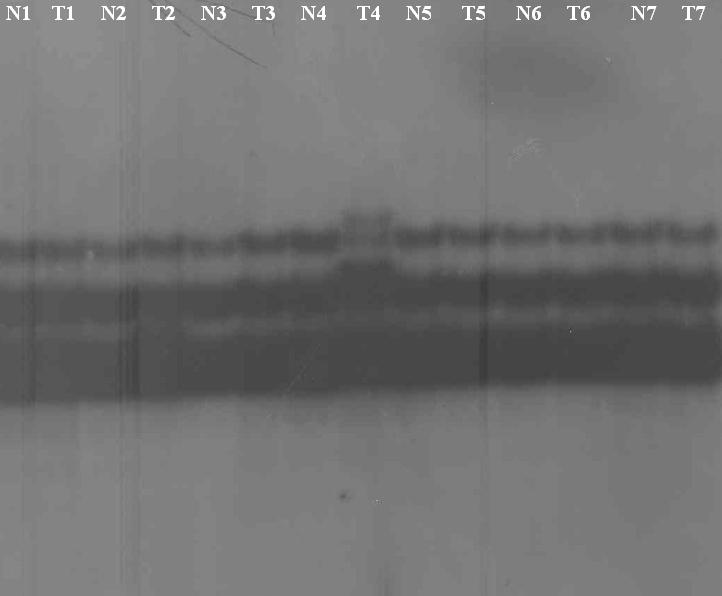

Supplement: Additional file 1 — Representative gel pictures of all the amplicons of the SMAD4 MCR (exon 2, 8-11) and KRAS exon 1 and also SSCP gel picture of KRAS exon 1 amplicons. [file 1471-2407-10-300-S1.DOC]
